# From Guidelines to Practice: A Lithium Monitoring Quality Improvement Project

**DOI:** 10.1192/bjo.2026.11157

**Published:** 2026-06-30

**Authors:** Esma Derouiche, Darren Sherrie, Shani Ross

**Affiliations:** NHS Lothian, Edinburgh, United Kingdom

## Abstract

**Aims:**

Lithium is a highly effective mood stabiliser but has a narrow therapeutic index. Inadequate monitoring increases the risk of toxicity, renal impairment and thyroiddys function. Consequently, NICE and local shared-care guidelines mandate structured baseline assessment and regular biochemical monitoring for all patients prescribed lithium.

This quality improvement (QI) project aimed to systematically evaluate adherence to NICE and local guideline standards for lithium monitoring within the inpatient rehabilitation service at the Royal Edinburgh Hospital (REH). The project sought to identify specific gaps in baseline and ongoing monitoring, assess the timeliness and completeness of required investigations. A further objective was to use these findings to inform targeted, sustainable interventions to improve compliance with monitoring standards, enhance patient safety and reduce the risk of preventable lithium-related adverse events within this high-risk population.

**Methods:**

A retrospective review was undertaken of all inpatients prescribed lithium across five rehabilitation wards at REH between July 2023 and July 2025. Ten patients met inclusion criteria. Data were extracted from electronic health records, the electronic prescribing system, laboratory results and Lothian Quality Improvement, Safety, Teaching, Supervision, Audit and Evaluation (QISTSAE) adverse event reports. Collected variables included patient demographics, lithium initiation, duration of treatment, the completion and timeliness of required monitoring investigations. Compliance was assessed against NICE guidance and local shared-care protocols.

**Results:**

Significant gaps in lithium monitoring were identified across the cohort. Urine albumin–creatinine ratio (uACR) testing, recommended for early detection of lithium-associated renal damage, was consistently absent in all patients reviewed. Serum calcium and thyroid function tests were missed in approximately 60% of cases. In addition, timeliness of monitoring was suboptimal, with several patients having overdue lithium levels, renal function tests or thyroid investigations. Renal function was not consistently rechecked following elevated lithium levels despite explicit guideline recommendations and over 10%of the results recorded supratherapeutic lithium concentrations during the review period, representing a clear safety concern.

**Conclusion:**

Lithium monitoring within the inpatient rehabilitation setting was inconsistent and frequently failed to meet guideline standards exposing patients to avoidable risk. uACR testing, calcium monitoring and thyroid surveillance represented particular areas of deficit. These findings highlight the need for system-level interventions to support safer lithium prescribing.

Planned next steps include expansion of the project to other inpatient and outpatient services, development of standardised lithium initiation and monitoring order sets in collaboration with laboratory services, targeted educational interventions for multidisciplinary teams to improve awareness, compliance and patient safety.

